# Murine GRXCR1 Has a Different Function Than GRXCR2 in the Morphogenesis of Stereocilia

**DOI:** 10.3389/fncel.2021.714070

**Published:** 2021-07-21

**Authors:** Chang Liu, Bo Zhao

**Affiliations:** Department of Otolaryngology-Head and Neck Surgery, Indiana University School of Medicine, Indianapolis, IN, United States

**Keywords:** hearing loss, hair cell, stereocilia, GRXCR1, GRXCR2

## Abstract

Mutations in human glutaredoxin domain-containing cysteine-rich protein 1 (*GRXCR1*) and its paralog *GRXCR2* have been linked to hearing loss in humans. Although both GRXCR1 and GRXCR2 are required for the morphogenesis of stereocilia in cochlear hair cells, a fundamental question that remains unclear is whether GRXCR1 and GRXCR2 have similar functions in hair cells. Previously, we found that GRXCR2 is critical for the stereocilia morphogenesis by regulating taperin localization at the base of stereocilia. Reducing taperin expression level rescues the morphological defects of stereocilia and hearing loss in *Grxcr2*-deficient mice. So far, functions of GRXCR1 in mammalian hair cells are still unclear. *Grxcr1*-deficient hair cells have very thin stereocilia with less F-actin content inside, which is different from *Grxcr2*-deficient hair cells. In contrast to GRXCR2, which is concentrated at the base of stereocilia, GRXCR1 is diffusely distributed throughout the stereocilia. Notably, GRXCR1 interacts with GRXCR2. In *Grxcr1*-deficient hair cells, the expression level of GRXCR2 and taperin is reduced. Remarkably, different from that in *Grxcr2*-deficient mice, reducing taperin expression level does not rescue the morphological defects of stereocilia or hearing loss in *Grxcr1*-deficient mice. Thus, our findings suggest that GRXCR1 has different functions than GRXCR2 during the morphogenesis of stereocilia.

## Introduction

As the fourth leading cause of disability, hearing loss is affecting over 5% of the population or 430 million people in the world, estimated by World Health Organization. Sensorineural hearing loss is the most common type of hearing loss, frequently caused by the damage of inner ear hair cells, which transform the mechanical sound stimuli into electrical signals (Gillespie and Muller, 2009; Pacentine et al., 2020). Many forms of hearing loss are due to genetic mutations. By 2011, 135 loci for the monogenic forms of human deafness had been reported (Richardson et al., 2011). So far, more than 60 of the affected genes have been identified, and many of those genes are specifically expressed in hair cells (Scheffer et al., 2015). Technological refinements in positional cloning and the sequencing of the human genome have dramatically accelerated the pace to identify deafness-related genes. However, the underlying mechanisms by which these genes affect auditory perceptions are only just beginning to be elucidated.

The glutaredoxin domain-containing cysteine-rich family of genes, *GRXCR1* and *GRXCR2*, have been identified as the mutated genes in nonsyndromic deafness DFNB25 and DFNB101, respectively (Odeh et al., 2010; Schraders et al., 2010; Imtiaz et al., 2014; Wonkam et al., 2021). Both GRXCR1 and GRXCR2 are specifically expressed in cochlear hair cells and essential for the morphogenesis of stereocilia, the mechanosensing subcellular organelles that protrude from the apical surface of hair cells (Beyer et al., 2000; Erven et al., 2002; Odeh et al., 2004, 2010; Avenarius et al., 2018; Liu et al., 2018). A previous study found that GRXCR2 is critical for the morphogenesis of stereocilia by regulating the localization of taperin at the base of stereocilia. Reducing taperin expression level rescues the morphological defects of stereocilia and partially restores the hearing in the *Grxcr2* mutant (Liu et al., 2018). Although the sequences of GRXCR1 and GRXCR2 are 32% identical, it is still unknown whether GRXCR1 and GRXCR2 have similar functions in hair cells.

The central region of GRXCR1 shows some similarity with glutaredoxin proteins, enzymes that reduce disulfide bonds or catalyze reversible protein glutathionylation or deglutathionylation. A previous study found that zebrafish GRXCR1 is essential for stereocilia morphogenesis through its glutaredoxin enzyme activity (Blanco-Sanchez et al., 2018). Interestingly, a significant amount of GRXCR1 is localized in the Golgi apparatus in zebrafish (Blanco-Sanchez et al., 2018), which is different from the localization of GRXCR1 in mouse hair cells (Odeh et al., 2010). Different localization indicates that GRXCR1 might have different functions in murine hair cells. The *bona fide* glutaredoxin enzyme activity of murine GRXCR1 needs experimental verification.

In this study, we analyzed the localization of GRXCR1 and GRXCR2 in hair cells. We reduced taperin expression level in *Grxcr1* mutant and characterized different mouse lines bearing *Grxcr1* and/or *taperin* mutations. In addition, we purified murine GRXCR1 protein and measured the glutaredoxin activity. Our results suggest that GRXCR1 has different functions than GRXCR2 during the morphogenesis of stereocilia.

## Materials and Methods

### Animal Models and Animal Care

*Grxcr2^−/−^* mouse (previously named as *Grxcr2^D46/D46^* mouse) and *taperin^−/−^* mouse (previously named as *taperin^in103/in103^* mouse) have been described previously (Liu et al., 2018). *Grxcr1^−/−^* mouse (also named as *Grxcr1^pi-2J^* mouse) was purchased from Jackson Laboratories, Bar Harbor, ME, USA (Odeh et al., 2010). All animal experiments were approved by Institutional Animal Care and Use Committee of Indiana University School of Medicine. Both male and female mice were used in our experiment. We did not find any sex-based differences.

### Scanning Electron Microscopy

The experiments were performed as described (Zhao et al., 2016; Liu et al., 2018). In brief, inner ears from P7 pups were dissected in a fixative containing 2.5% glutaraldehyde and 4% formaldehyde. Samples were then fixed for 1 h at RT and dissected to remove the stria vascularis, Reissner’s membrane, and tectorial membrane. After being post-fixed in the same fixative overnight at 4°C, samples were fixed with 1% OsO_4_ for 1 h at room temperature. Then samples were serially dehydrated in ethanol, dried in a critical point drier (Autosamdri-815A, Tousimis, Rockville, MD, USA), finely dissected, and mounted on aluminum stubs. Samples were then coated by gold. Images were captured using a JEOL 7800F scanning electron microscope (Jeol, Tokyo, Japan). At least three animals representative of each experimental paradigm were analyzed.

To measure the width of the tallest row of stereocilia in inner hair cells, single hair cells were imaged at high magnification (×~20,000) using a JEOL 7800F scanning electron microscope. Then, the width of the tallest row of stereocilia at about 1/3 of the height from the top was measured using ImageJ (NIH, Bethesda, MD, USA).

### Whole Mount Immunostaining

Whole mount staining was carried out as described (Zhao et al., 2016; Liu et al., 2018). In brief, organ of Corti tissue was dissected and fixed in a fixative containing 4% paraformaldehyde (PFA) for 20 min. Samples were blocked for 20 min at room temperature in a blocking buffer containing 5% bovine serum albumin (BSA), 1% goat serum, and 0.5% Triton X-100. Then, samples were incubated with primary antibodies overnight at 4°C. Samples were washed and incubated 2 h at room temperature with secondary antibodies. After mounting in ProLong^®^ Antifade Reagents (Invitrogen, Waltham, MA, USA), stacked images were captured by deconvolution microscope (Leica) using a ×100 objective. Images were deconvoluted using blind deconvolution method.

To costain β-actin and γ-actin, cochlear whole mounts were fixed in 4% PFA for 20 min and then ice-cold methanol for 15 min. Then, samples were blocked for 20 min at room temperature in a blocking buffer containing 5% BSA, 1% goat serum, and 0.5% Triton X-100. Then, samples were incubated with a primary antibody against γ-actin (Abcam, Cambridge, UK) overnight at 4°C. Samples were then washed and incubated with secondary antibody for 2 h at room temperature. Then, samples were washed again and incubated with FITC-conjugated β-actin antibody (Abcam, Cambridge, UK) overnight at 4°C. After washing, samples were mounted and imaged.

To raise antibodies against GRXCR1, two peptides (NEQEKDQDNLLVLART and KFEEKNIALNGDYGKELDER) were covalently linked to KLH and immunized New Zealand rabbits (Thermo Fisher Scientific, Waltham, MA, USA). Affinity purification was then performed. Other primary antibodies used in this study were as follows: anti-GRXCR2 (Cat# HPA059421, Sigma, Saint Louis, MO, USA) and anti-taperin (Cat# HPA020899, Sigma, Saint Louis, MO, USA). Additional reagents were as follows: Alexa Fluor 488-phalloidin (Thermo Fisher Scientific, Waltham, MA, USA), Alexa Fluor 568-phalloidin (Thermo Fisher Scientific, Waltham, MA, USA), Alexa Fluor 647-phalloidin (Thermo Fisher Scientific, Waltham, MA, USA), Alexa Fluor 488 goat anti-rabbit (Thermo Fisher Scientific, Waltham, MA, USA), Alexa Fluor 555 goat anti-mouse (Thermo Fisher Scientific, Waltham, MA, USA), and Alexa Fluor 546 goat anti-rabbit (Thermo Fisher Scientific, Waltham, MA, USA).

### Auditory Brainstem Response Measurement

Auditory brainstem responses (ABRs) of mice were recorded as described (Zhao et al., 2016; Liu et al., 2018) using TDT Bioacoustic system 3 and BioSigRZ software. In brief, mice were anesthetized using a mixture of 100 mg/kg ketamine and 10 mg/kg xylazine. Electrodes were inserted under the skin at the vertex and ipsilateral ear, while a ground was inserted under the skin near the tail. The speaker was placed 5 cm away from the mouse ear, and tone stimulus is presented 21 times per second. Band pass filtered from 300–3,000 Hz and a total of 512 responses were averaged at each frequency and level combination. The intensity of sound stimulus was started at 90 dB sound pressure level (SPL) and decreased to 10 dB SPL stepwise to a sub-threshold level. ABR thresholds were analyzed for a range of frequencies (for pure tone, 4–28 kHz). If no ABR wave was detected at maximum intensity stimulation, a nominal threshold of 90 dB was assigned.

### Cell Culture

HEK293 cell line was obtained from ATCC (Manassas, VA, USA). Cells were maintained in the DMEM medium (Thermo Fisher Scientific, Waltham, MA, USA) supplemented with 10% heat-inactivated fetal bovine serum (Thermo Fisher Scientific, Waltham, MA, USA), 100 U/ml penicillin, and 100 μg/ml streptomycin (Thermo Fisher Scientific, Waltham, MA, USA). Cells were grown at 37°C in a 5% CO_2_-humidified atmosphere.

### cDNA Constructions, Immunoprecipitations, and Western Blots

The coding sequence of *Grxcr1* was amplified from mouse cochlear cDNA library. Expression of the constructs, immunoprecipitations, and western blots were carried out as described (Senften et al., 2006; Zhao et al., 2016; Liu et al., 2018). Immunoprecipitation experiments were carried out at least three times to verify the reproducibility of the data. The following antibodies were used for the experiments: anti-Myc (Cat# 2278S, Cell Signaling Technology, Danvers, MA, USA), anti-Myc (Cat# sc-40, Santa Cruz Biotechnology, Dallas, TX, USA), and anti-GFP (Cat# sc-9996, Santa Cruz Biotechnology, Dallas, TX, USA).

### Purification of GRXCR1

Mouse *Grxcr1* cDNA fused with six XHis tag at C terminus was inserted into pMAL-c5x plasmid. *Escherichia coli* BL21 (DE3) cells were cultured in LB containing 100 μg/ml ampicillin at 37°C. The expression of fusion protein was induced overnight with 1 mM isopropylthio-β-galactoside at room temperature. Cells were harvested and resuspended in 25 mM Tris-base (pH 7.6) and 125 mM NaCl buffer. After sonication, the lysate was centrifuged at 15,000 rpm for 30 min. The supernatant was incubated with His-bind resin, and then, protein was eluted by an elution buffer containing 250 mM imidazole. To increase the purity, the eluted protein was then incubated with amylose resin and eluted by an elution buffer containing 10 mM maltose.

### Glutaredoxin Activity Assay

Glutaredoxin activity assay was performed following the published protocol (Mieyal et al., 1991a, b). In brief, the 0.9 ml reaction mixture contained 0.2 mM NADPH, 0.5 mM GSH, 0.1 M potassium phosphate buffer (pH 7.4), 0.4 U of GSSG reductase, and an aliquot of purified GRXCR1 or glutaredoxin-2 (GLRX2; Thermo Fisher Scientific, Waltham, MA, USA). The mixture was preincubated for 5 min at 30°C. After adding 0.1 ml 20 mM hydroxyethyl disulfide (HEDS) to initiate the reaction, *A*_340 nm_ was measured by Synergy H1 spectrophotometer (BioTek, Winooski, VT, USA) to determine the slope of the linear portion of the time course for absorption loss (subtracted blank control). One unit of glutaredoxin activity was defined as 1 μmol of NADPH oxidized per minute under these standard assay conditions.

### Quantification and Statistical Analysis

All data are mean ± standard error of the mean (SEM). Student’s two-tailed unpaired *t* test or two-way ANOVA were used to determine statistical significance (*p < 0.05, **p < 0.01, ***p < 0.001).

## Results

### GRXCR1 is Essential for Stereocilia Morphogenesis

**Figure 1:**
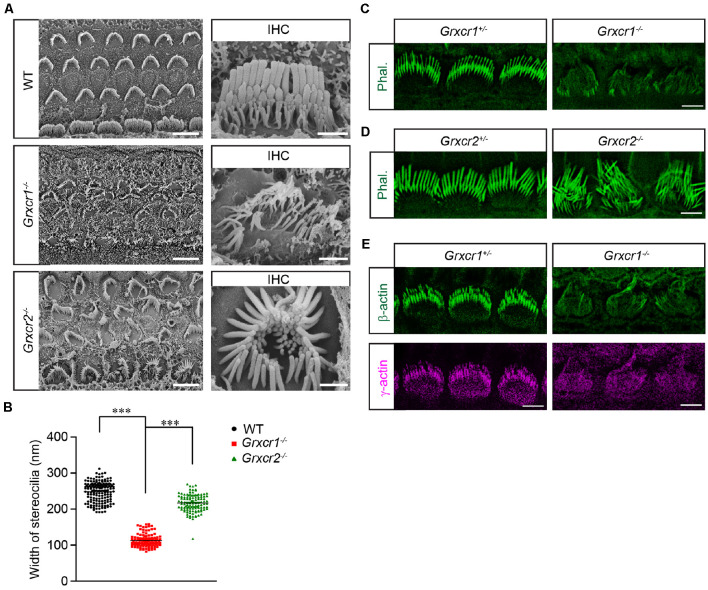
Stereocilia morphology in *Grxcr1*-deficient hair cells. **(A)** Scanning electron microscope images showing auditory epithelia of wild-type, *Grxcr1^−/−^*, and *Grxcr2^−/−^* mice at the age of P7. Stereocilia of inner hair cells (IHCs) and outer hair cells (OHCs) from *Grxcr1^−/−^* and *Grxcr2^−/−^* hair cells were disorganized. Note, stereocilia in *Grxcr1^−/−^* hair cells were extremely thin. Scale bars: left, 5 μm; right 1 μm. **(B)** Width of the tallest row of stereocilia of IHCs from wild-type, *Grxcr1^−/−^*, and *Grxcr2^−/−^* mice at the age of P7. All values are represented as the mean ± SEM. ***p < 0.001 by Student’s t test. **(C,D)** Cochlear whole mounts from *Grxcr1^−/−^* **(C)** and *Grxcr2^−/−^* **(D)** mice at P7 were stained for phalloidin to reveal stereocilia. Note the weak phalloidin staining signal in *Grxcr1^−/−^* IHCs. Scale bars: 5 μm. **(E)** Cochlear whole mounts from *Grxcr1^−/−^* mice at P7 were stained for β-actin and γ-actin. Scale bars: 5 μm.

Both GRXCR1 and GRXCR2 are required for stereocilia morphogenesis and auditory perception. To investigate whether their detailed functions are similar in hair cells, we characterized the *Grxcr1* null mutant (*Grxcr1^pi-2J^*, referred as *Grxcr1^−/−^* hereafter) and *Grxcr2* null mice that we previously generated (*Grxcr2^D46/D46^*, referred as *Grxcr2^−/−^* hereafter). Both *Grxcr1^−/−^* and *Grxcr2^−/−^* hair bundles are severely disorganized and have lost the typical staircase organization at postnatal day 7 (P7). Hair bundle fragmentation and misorientation are frequently observed in hair cells from both mutants. Remarkably, stereocilia in *Grxcr1^−/−^* hair cells are extremely thin compared with that in the wild-type hair cells or *Grxcr2^−/−^* hair cells (Figures 1A,B), which is consistent with the previous results (Beyer et al., 2000; Erven et al., 2002; Odeh et al., 2004). Stereocilia are filled with tightly cross-linked and uniformly oriented actin filaments that provide stiffness. The very strong phalloidin staining signal in the wild-type stereocilia suggests the abundance of actin filaments inside the stereocilia. Remarkably, very low phalloidin staining signal along the disorganized stereocilia was obtained from *Grxcr1^−/−^* mice, suggesting that the F-actin content is dramatically reduced in the *Grxcr1^−/−^* hair bundles (Figure 1C). Although stereocilia are also disorganized in the *Grxcr2^−/−^* mice, there is no detectable reduction of F-actin content inside the stereocilia, revealed by phalloidin staining (Figure 1D). To further confirm this result, the amount of β-actin and γ-actin, two major actin isoforms in the stereocilia, was evaluated in *Grxcr1^−/−^* hair cells by immunostaining. Both β-actin and γ-actin are reduced in the *Grxcr1^−/−^* hair cells (Figure 1E). These results suggest that the pathophysiological changes in hair cells associated with *Grxcr1* and* Grxcr2* mutations are different.

### GRXCR1 is Diffused Along the Stereocilia

**Figure 2:**
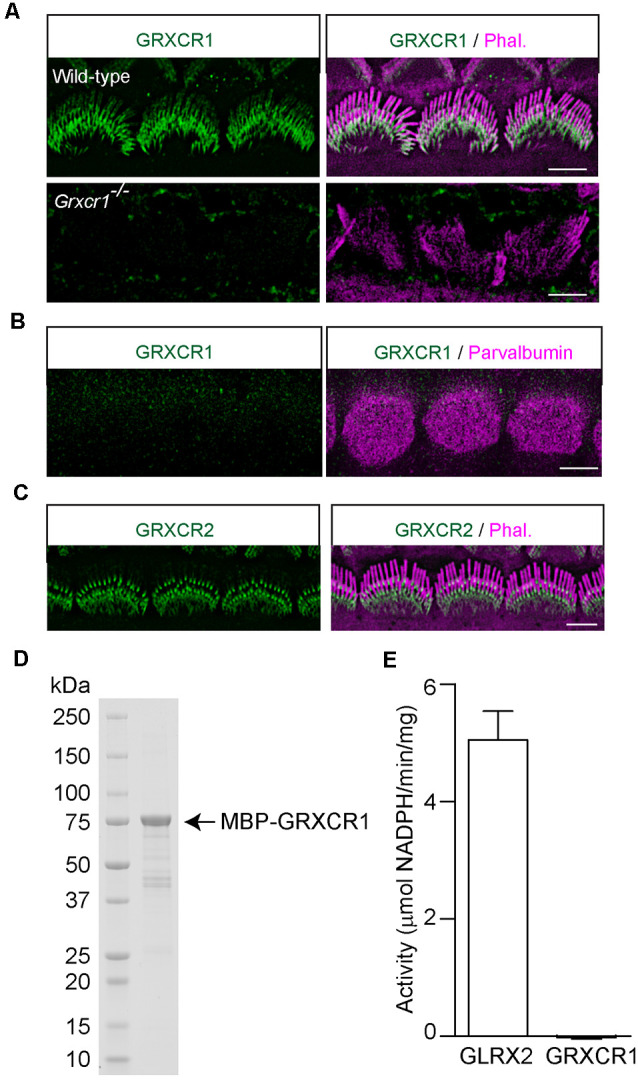
Localization of GRXCR1 and GRXCR2 in hair cells. **(A)** Costaining of P7 cochlear whole mounts with GRXCR1-antibody (green) and phalloidin (magenta). Note the absence of a signal in *Grxcr1^−/−^* inner hair cells. **(B)** Costaining of P7 wild-type cochlear whole mounts with GRXCR1-antibody (green) and parvalbumin-antibody (magenta) to reveal cell body of inner hair cells. Note that there is no signal of GRXCR1 in the cell body. **(C)** Costaining of P7 wild-type cochlear whole mounts with GRXCR2-antibody and phalloidin. Note, GRXCR2 is concentrated at the base of stereocilia. **(D)** GRXCR1 was expressed and purified from *E. coli*. **(E)** Glutaredoxin activity of GLRX2 and GRXCR1 was measured. Note that there is no detectable enzyme activity of GRXCR1. Scale bars: 5 μm.

Odeh et al. (2010) in Dr. David Kohrman’s lab found that GRXCR1 is diffused along the stereocilia in murine hair cells. In zebrafish, a significant amount of GRXCR1 is also localized in the Golgi apparatus and endoplasmic reticulum—Golgi intermediate compartment (Odeh et al., 2010; Blanco-Sanchez et al., 2018). In our previous studies, we found that exogenously expressed GRXCR1 is localized along the stereocilia without any obvious immunostaining signals in ER or Golgi structures (Liu et al., 2018). To study the localization of endogenous GRXCR1 protein in murine hair cells, we generated an antibody against GRXCR1. Immunostaining was performed using cochlear whole mounts dissected from wild-type and *Grxcr1^−/−^* mice. The immunostaining signal outlined the shape of the stereociliary bundle in the wild-type cochlea, but not in the *Grxcr1*-deficient cochlea (Figure 2A), suggesting that the antibody specifically recognize GRXCR1 in murine hair cells. In agreement with the findings of Odeh et al. (2010), GRXCR1 was localized along the shaft of stereocilia in hair cells (Figure 2A). Interestingly, we also noticed a stronger immunostaining signal at the base of stereocilia especially in inner hair cells, suggesting that GRXCR1 is relatively concentrated at the base of stereocilia (Figure 2A). In addition, we did not see any immunostaining signal of GRXCR1 in the cell body (Figure 2B), which is different from that in zebrafish hair cells (Blanco-Sanchez et al., 2018). Different from GRXCR1, GRXCR2 is localized at the base of stereocilia (Figure 2C).

In zebrafish, GRXCR1 is essential for the morphogenesis of stereocilia through its glutaredoxin activity (Blanco-Sanchez et al., 2018). Although the central region of mammalian GRXCR1 shows some similarity with glutaredoxin proteins, it lacks the typical dual cysteines essential for enzyme activity, and the putative thioredoxin fold in mammalian GRXCR1 might just act as an interface mediating the protein–protein interaction (Odeh et al., 2010). To investigate whether murine GRXCR1 has enzyme activity, GRXCR1 was expressed in *E. coli* and purified (Figure 2D). Catalytic properties of murine GRXCR1 were determined in parallel with and compared with human GLRX2. Consistent with previous studies, GLRX2 showed high glutaredoxin activity (Gladyshev et al., 2001); however, we did not detect any enzyme activity of murine GRXCR1 (Figure 2E), suggesting that in murine hair cells, GRXCR1 is essential for the morphogenesis of stereocilia probably *via* different mechanisms.

### Localization of Taperin and GRXCR2 in *Grxcr1*-Deficient Hair Cells

**Figure 3:**
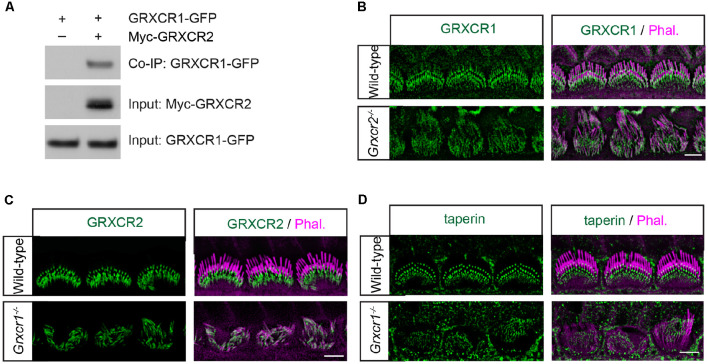
Localization of taperin and GRXCR2 in *Grxcr1*-deficient hair cells. **(A)** HEK293 cells were transfected with GRXCR1-GFP and Myc-GRXCR2. Immunoprecipitation was carried out with Myc-antibody. The upper row shows co-IP result and the lower rows show input proteins. **(B)** Cochlear whole mounts from *Grxcr2^−/−^* mice at P7 was stained for GRXCR1. **(C,D)** Cochlear whole mounts from *Grxcr1^−/−^* mice at P7 were stained for GRXCR2 **(C)** and taperin **(D)**. Scale bars: 5 μm.

A previous study found that GRXCR1 interacts with GRXCR2 when they are expressed in yeast (Avenarius, 2012). To investigate whether GRXCR1 interacts with GRXCR2 in mammalian cells, we carried out co-immunoprecipitation experiments with extracts from HEK293 cells that were transfected with Myc-tagged GRXCR2 and GFP-tagged GRXCR1. Indeed, GRXCR1-GFP was co-immunoprecipitated with Myc-GRXCR2 (Figure 3A), suggesting a heterodimeric interaction between GRXCR1 and GRXCR2.

To investigate the extent to which the interaction between GRXCR1 and GRXCR2 is required for their proper localization in the stereocilia, immunohistochemistry experiments were performed. In the *Grxcr2^−/−^* hair cells, GRXCR1 was a little bit more diffusely distributed along the stereocilia (Figure 3B). In the *Grxcr1^−/−^* hair cells, GRXCR2 was diffused along the stereocilia and the intensity of immunostaining signal was reduced (Figure 3C). The intensity of taperin immunostaining signal was also reduced, and no diffusion of taperin along the stereocilia was observed in P7 *Grxcr1^−/−^* hair cells (Figure 3D). As the stereocilia in *taperin^−/−^* hair cells are fairly normal at P7 (Liu et al., 2018), the extremely thin stereocilia in *Grxcr1^−/−^* hair cells are likely not mainly caused by the reduction of taperin. Since the stereocilia are extremely thin in *Grxcr1^−/−^* hair cells, it is possible that the reduction of taperin and GRXCR2 might be secondary to the stereociliary morphogenesis defects.

### Reducing Taperin Expression Could Not Rescue the Morphological Defects of Stereocilia in *Grxcr1*-Deficient Mice

**Figure 4:**
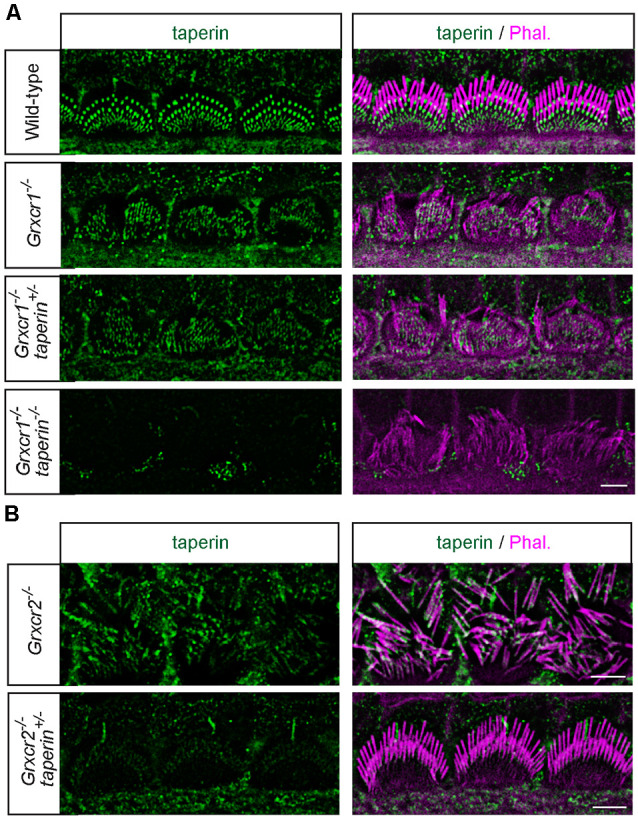
Analysis of stereocilia morphology by immunostaining. **(A)** Whole mounts from the middle region of the cochlea from wild-type, *Grxcr1^−/−^*, *Grxcr1^−/−^*taperin^+/–^, and *Grxcr1^−/−^*taperin^−/−^ mice at the age of P7 were stained with taperin-antibody (green) and phalloidin (magenta). Note the disorganized stereocilia in *Grxcr1^−/−^*, *Grxcr1^−/−^*taperin^+/–^, and *Grxcr1^−/−^*taperin^−/−^ mice. **(B)** Whole mounts from the middle region of cochlea from *Grxcr2^−/−^* and *Grxcr2^−/−^*taperin^+/–^ mice at the age of P7 were stained with taperin-antibody and phalloidin. Note the disorganized stereocilia in *Grxcr2^−/−^* mice and well-maintained stereocilia in *Grxcr2^−/−^*taperin^+/–^ mice. Scale bars: 5 μm.

The morphological defects of stereocilia in *Grxcr2^−/−^* mice is caused by the mislocalization of taperin, as reducing taperin expression level rescues the stereocilia morphology in *Grxcr2^−/−^*-deficient hair cells (Liu et al., 2018). The pathophysiological changes in hair cells associated with *Grxcr1* and* Grxcr2* mutations are different, suggesting that *Grxcr1* deficiency induced the stereocilia disorganization might be through a different mechanism. GRXCR1 interacts with GRXCR2. Lack of GRXCR1 affects the localization and expression of GRXCR2 and taperin in stereocilia. To investigate whether the morphological defects in *Grxcr1^−/−^* hair cells is caused or partially caused by taperin, we crossed *Grxcr1^−/−^* mice with *taperin* null mice that we previously generated (*taperin^in103/in103^*, referred as *taperin^−/−^* hereafter) and then immunostaining was performed. Similar to *Grxcr1^−/−^* hair cells, *Grxcr1^−/−^*taperin^+/–^ and *Grxcr1^−/−^*taperin^−/−^ hair cells have disorganized stereocilia with less F-actin content inside (Figure 4A). In contrast, reducing taperin expression level rescued the morphological defects of stereocilia in *Grxcr2^−/−^* hair cells (Figure 4B).

**Figure 5:**
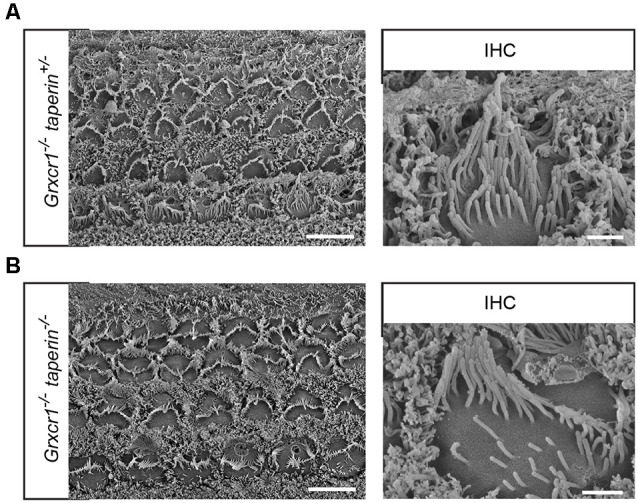
Analysis of stereocilia morphology by scanning electron microscopy. **(A,B)** Whole mounts from the middle region of the cochlea from *Grxcr1^−/−^taperin^+/–^* **(A)** and *Grxcr1^−/−^*taperin^−/−^ **(B)** mice at the age of P7 were analyzed by scanning electron microscopy. Note, stereocilia in *Grxcr1^−/−^*, *Grxcr1^−/−^*taperin^+/–^, and *Grxcr1^−/−^*taperin^−/−^ mice were extremely thin and disorganized. Scale bars: left, 5 μm; right, 1 μm.

To analyze the stereocilia morphology in more detail, we carried out additional scanning electron microscopy analyses with hair cells at P7. Similar to that in *Grxcr1^−/−^* hair cells, stereocilia in *Grxcr1^−/−^*taperin^+/–^ and *Grxcr1^−/−^*taperin^−/−^ hair cells are very thin and disorganized (Figure 5). These results suggest that reducing taperin expression level could not restore the morphological defects in *Grxcr1^−/−^* hair cells.

### Reducing Taperin Expression Could Not Restore the Hearing in *Grxcr1*-Deficient Mice

**Figure 6:**
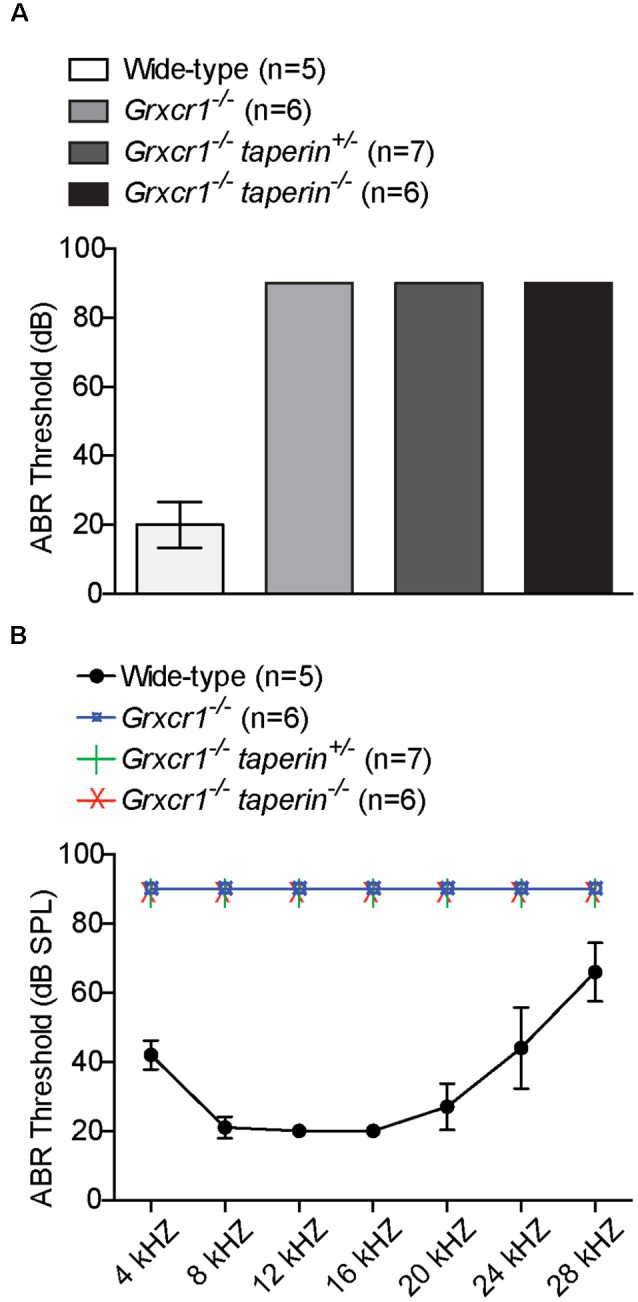
Analysis of hearing function by ABR. **(A,B)** ABR thresholds for click stimuli **(A)** and pure tones **(B)** at the age of 6 weeks. Note the profound hearing loss in *Grxcr1^−/−^*, *Grxcr1^−/−^*taperin^+/–^, and *Grxcr1^−/−^*taperin^−/−^ mice across the entire frequency spectrum analyzed. *p* < 0.001 between wild-type and *Grxcr1^−/−^*, *Grxcr1^−/−^*taperin^+/–^, and *Grxcr1^−/−^*taperin^−/−^ mice (two-way ANOVA).

To investigate whether reducing taperin expression could restore the hearing in *Grxcr1^−/−^* mice, we measured auditory perceptions of 6-week-old *Grxcr1^−/−^*taperin^+/–^ and *Grxcr1^−/−^*taperin^−/−^ mice. Similar to the *Grxcr1^−/−^* mice, *Grxcr1^−/−^*taperin^+/–^ and *Grxcr1^−/−^*taperin^−/−^ could not respond to the 90-dB sound stimuli, suggesting that they were profoundly deaf (Figure 6A). Recording ABRs in response to pure tones revealed that all these mice had profound hearing loss across the entire analyzed frequency spectrum (Figure 6B). These results suggest that reducing taperin expression level could not restore the hearing in the *Grxcr1^−/−^* mice. Thus, our results suggest that GRXCR1 has different functions than GRXCR2 during the morphogenesis of stereocilia.

## Discussion

GRXCR1 and GRXCR2 have more than 30% amino acid identity. Both of them are essential for the morphogenesis of stereocilia and auditory perception. However, several lines of evidence in this study suggest that they have different functions in hair cells. First, the pathophysiological changes of stereocilia associated with *Grxcr1* and *Grxcr2* mutations are very different. Second, although both of them are localized at the stereocilia, GRXCR1 is diffusely distributed throughout the stereocilia. Third, although we could alleviate of the auditory defects in *Grxcr2^−/−^* mice by reducing taperin expression level, using a similar strategy, we could not rescue the morphological defects of stereocilia or restore the hearing in *Grxcr1^−/−^* mice.

GRXCR1 shows some similarity with glutaredoxin proteins. In zebrafish, the glutaredoxin activity of GRXCR1 is crucial for regulating the physical interaction between Harmonin and Sans (Blanco-Sanchez et al., 2018). Consistently, *Grxcr1*, *harmonin*, and *sans* zebrafish mutants have very similar phenotypes with significantly thinner, fewer hair bundles and splayed stereocilia (Blanco-Sanchez et al., 2018). However, in mouse, the phenotypes of *Grxcr1*, *harmonin*, and *sans*-mutant hair cells are different. *Grxcr1* mutant hair cells have very thin and short stereocilia, while the length and width of the tallest row of stereocilia in *harmonin* and *sans* mutant hair cells are similar to the wild-type hair cells (Lefevre et al., 2008; Corns et al., 2018), suggesting that GRXCR1 probably has different functions in mouse hair cells. In our study, we purified mouse GRXCR1 and measured its glutaredoxin activity. We did not detect any enzyme activity using purified murine GRXCR1. There is a possibility that GRXCR1 purified from bacteria might not fold properly. Thus, the *bona fide* glutaredoxin activity of murine GRXCR1 needs to be further verified using other experimental approaches in future. GRXCR1 also shows some similarity with THRUMIN1 from *Arabidopsis thaliana* (Avenarius, 2012). An *in vitro* study shows that THRUMIN1 binds to and bundles F-actin (Whippo et al., 2011). Interestingly, in the *Grxcr1^−/−^* hair cells, the stereocilia are extremely thin and F-actin content is reduced. The question arose whether GRXCR1 is essential for the morphogenesis of stereocilia by directly or indirectly regulating actin cytoskeleton inside the stereocilia. To test this hypothesis, further studies to investigate whether purified GRXCR1 could bind to actin and regulate actin dynamics *in vitro* would be informative.

Although GRXCR1 and GRXCR2 have similar amino acid sequences, only GRXCR2 binds to taperin (Liu et al., 2018). In addition, different localization patterns of these two proteins in stereocilia also suggest that they probably have different binding partners in hair cells. To extensively illustrate the functions of GRXCR1 and mechanisms of *Grxcr1* deficiency-induced hearing loss, it will be of interest to screen interacting proteins of GRXCR1 and investigate the extent to which those binding partners are required for GRXCR1 functions and stereocilia morphogenesis in hair cells.

## Data Availability Statement

The original contributions presented in the study are included in the article, further inquiries can be directed to the corresponding author.

## Ethics Statement

The animal study was reviewed and approved by Institutional Animal Care and Use Committee of Indiana University School of Medicine.

## Author Contributions

CL and BZ made significant contributions in the methodology, investigation, and writing. BZ made significant contributions in the conceptualization and supervision of the study. All authors contributed to the article and approved the submitted version.

## Conflict of Interest

The authors declare that the research was conducted in the absence of any commercial or financial relationships that could be construed as a potential conflict of interest.
